# Verification of patient‐setup accuracy using a surface imaging system with steep measurement angle

**DOI:** 10.1002/acm2.13872

**Published:** 2022-12-20

**Authors:** Fumihiro Sasaki, Yuusuke Yamashita, Satoshi Nakano, Masayori Ishikawa

**Affiliations:** ^1^ Teine Keijinkai Hospital Sapporo Hokkaido Japan; ^2^ Graduate School of Biomedical Science and Technology Hokkaido University Sapporo Hokkaido Japan; ^3^ Faculty of Health Sciences Hokkaido University Sapporo Hokkaido Japan

**Keywords:** setup error, skin marker, surface‐guided radiation therapy, tomotherapy

## Abstract

**Purpose:**

We evaluate an SGRT device (Voxelan HEV‐600 M/RMS) installed with Radixact, with the view angle of the Voxelan's camera at 74 degrees. The accuracy of Voxelan with this steep angle was evaluated with phantom experiments and inter‐fractional setup errors of patients.

**Methods:**

In the phantom experiments, the difference between the measured values of Voxelan from the truth was evaluated for translations and rotations. The inter‐fractional setup error between the setup using skin markers with laser localizer (laser setup: LS) and the setup using Voxelan (surface setup: SS) was compared for head and neck (*N* = 19), chest (*N* = 7) and pelvis (*N* = 9) cases. The inter‐fractional setup error was calculated by subtracting from bone matching by megavoltage computed tomography (MVCT) as ground truth.

**Results:**

From the phantom experiments, the average difference between the measured values of Voxelan from the truth was within 1 mm and 1 degree. In all cases, inter‐fractional setup error based on MVCT was not significantly different between LS and SS by Welch's *t*‐test (*P* > 0.05). The vector offset of the LS for head and neck, chest, and pelvis were 6.5, 9.6, and 9.6 mm, respectively, and that of the SS were 5.8, 8.6, and 12.6 mm, respectively. Slight improvement was observed for the head and neck, and chest cases, however, pelvis cases were not improved because the umbilical region could not be clearly visualized as a reference.

**Conclusion:**

The results show that SS in Voxelan with an installation angle of 74 degrees is equal to or better than LS.

## 1 INTRODUCTION

The accuracy of dose administration to treated patients in radiation therapy is described in ICRU Report 24.^1^ Among the recommendations, the total uncertainty of the dose to the patient in radiation therapy should be 5% or less. Furthermore, the spatial position uncertainty due to geometric patient movement and device accuracy should be 10 mm or less.^1^ In order to maintain the spatial position uncertainty of 10 mm, it is necessary to improve the setup accuracy. Setup for radiation therapy using skin markers is the most common method.^2^, ^3^, ^4^ However, the skin markers must be preserved throughout the entire treatment period and should be performed with the consent and cooperation of the patient, such as not to wash it off when bathing.^2^ Problems of accuracy deterioration due to the fading of skin markers have also been pointed out.^4^ On the other hand, semi‐permanent tattoos can adversely affect long‐term cosmetic problems, which in turn can have psychological consequences in terms of aesthetics.^2^, ^3^, ^4^

Surface guided radiation therapy (SGRT) has recently been clinically used as a new image‐guided radiotherapy (IGRT) modality that performs position matching based on the position information of the patient's surface. It has been reported that SGRT improves the position reproducibility of patients who receive radiation in areas surrounded by superficial and soft tissues, such as breast cancer, compared to conventional IGRT based on bone structure.^5^ Cosmetic considerations can also be achieved by reducing the range and amount of skin markers on the body surface, reducing the social and psychological burden on breast cancer patients.^6^

Radixact (Accuray Inc., CA, USA) can perform patient registration in 3D using megavoltage computed tomography (MVCT), the patient position can be corrected remotely in lateral, longitudinal, vertical, and roll directions. However, if large misalignments in the yaw and pitch directions are detected, at least 5 min of additional time is required to repositioning of the patient with MVCT acquisition. When the planning target volume (PTV) is along the body axis, such as in cases of esophageal cancer, the effects of pitch and yaw cannot be ignored and may cause setup errors. MVCT imaging often requires re‐setup which leads to an extension of treatment time; however, using an SGRT device makes it easier to correct the patient position for pitch and yaw, and throughput can be improved by reducing the number of MVCT acquisitions.^7^, ^8^ In Japan, there are few reports on the introduction of SGRT devices into ring‐type linear accelerators, and the combination of Radixact and Voxelan HEV‐600 M/RMS (Voxelan. ERD Co., Okayama, Japan), which is a single‐camera SGRT device, has not yet been reported.

The purpose of this study was to verify the accuracy of Voxelan at an installation angle of 74 degrees using a phantom, and compare the inter‐fractional setup errors when using a laser and skin markers for the initial setup of patients and when using Voxelan. The feasibility of initial patient setup using Voxelan was verified.

## 2 METHODS AND MATERIALS

**FIGURE 1:**
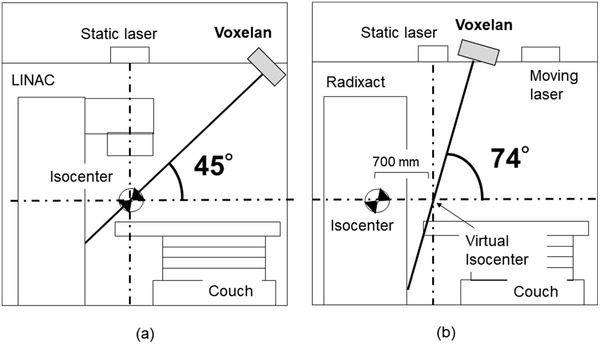
Schematic illustrations of camera geometry in the Voxelan system: (a) Conventional LINAC and (b) Radixact

When used in combination with a conventional C‐type linear accelerator (Linac), Voxelan is mounted on the ceiling of the treatment room where the patient's body surface can be sufficiently detected even during treatment, and the isocenter is captured from an angle of 45 degrees (Figure 1a). However, the treatment section of the Radixact is inside the bore of the main body, and a virtual isocenter is defined at a position 700 mm in front of the treatment location. Setup is performed at the virtual isocenter (Figure 1b). In addition, Radixact cannot be monitored at the treatment position because the bed moves during treatment. For these reasons, Voxelan was installed toward the virtual isocenter and operated for positioning the initial setup. However, in our treatment room, Voxelan could not be installed at the installation reference angle of 45 degrees because it interferes with the moving laser attached to the Radixact. Voxelan was therefore installed at a position where the virtual isocenter can be captured at an angle of 74 degrees from the ceiling, where it does not interfere with the moving laser (Figure 1b).

After confirming the position accuracy of Voxelan at the installation angle of 74 degrees, we compared and evaluated inter‐fractional setup error when the setup was performed using a laser and skin markers and inter‐fractional setup error when the setup is performed using the image information of the patient surface by Voxelan. The equipment used and specific methods are described below.

### 2.1 Description of Voxelan

**FIGURE 2:**
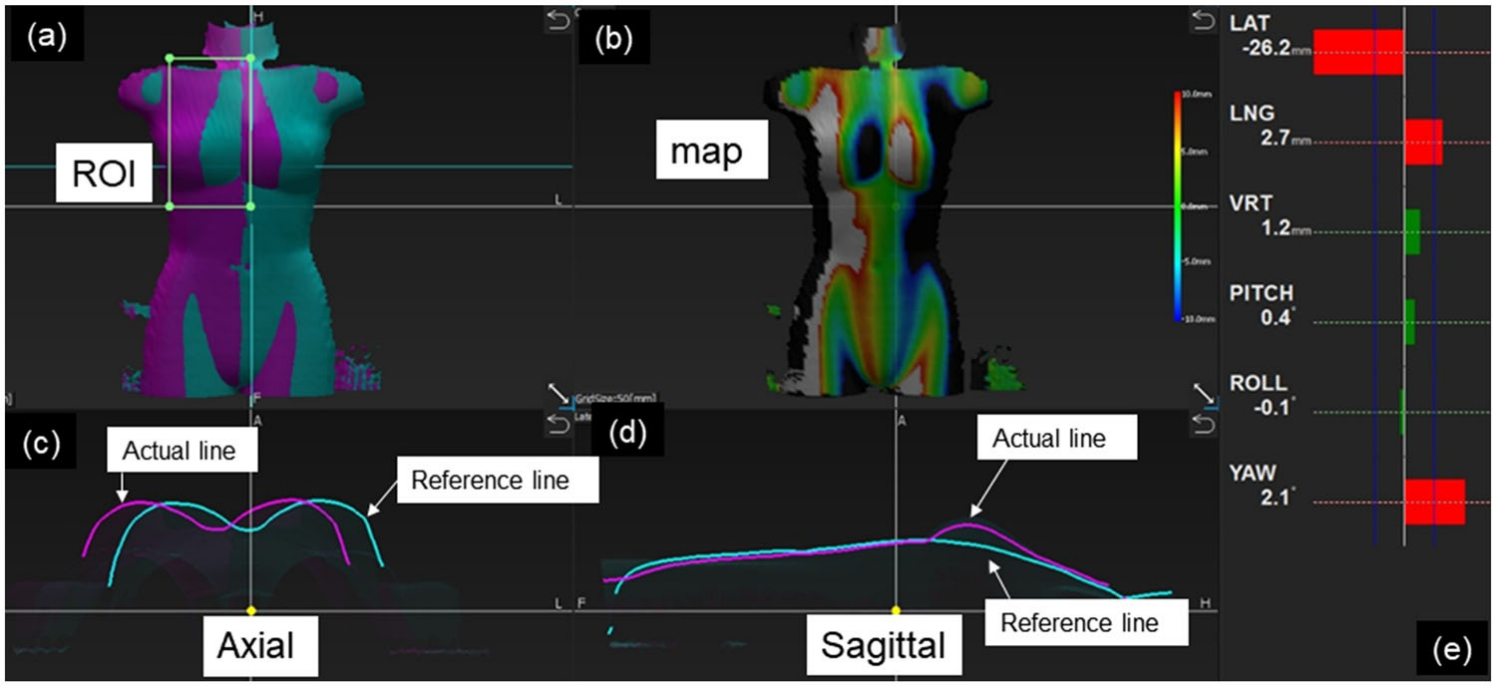
Screen capture image of the Voxelan software. (a) Overlay of measured and reference data. ROI setting is performed on this pane, (b) difference color map between measured and reference data, (c) axial profiles of measured and reference data, (d) sagittal profiles of measured and reference data, and (e) translational and rotational differences from the reference data

Voxelan constructs 3D data by irradiating the patient's body surface with line laser light and acquiring reflected light with a single camera (Table 1). With this technology, it can perform positioning at the time of setup. As shown in Table 1, the Voxelan has a field of view of 600 mm × 450 mm × 600 mm, and measurement is performed in two frames per seconds (including laser scanning and data output) with a camera resolution of 1280 × 1024 pixels. The surface depth data for the vrt direction is stored in 16‐bit data in the matrix of 400 (lat) × 300 (lng). The resolution for lat and lng is 1.5 mm and that for vrt is 0.023 mm which is 64 times higher than lat and lng. Figure 2 shows the display screen of Voxelan. The display screen shows body surface data constructed by scanning, a difference image between body surface data and reference data, each cross‐sectional profile of body surface data and reference data, and the measurement result of the position error in 6‐axis directions in the region of interest (ROI). There are two ways to create reference data: one constructed from CT images acquired by planning kilo voltage computed tomography (kVCT), defined as the kVCT reference; and one constructed from the scanned image of the Voxelan, defined as the surface reference.

**TABLE 1:** Performance overview of commercially available SGRT monitoring systems fps, frames per second; px, pixel

**System** **(vendor)**	**Optical technology**	**Treatment unit#** **hardware**	**Field‐of‐view** **(Lat × Long × Vert)**	**Camera** **resolution**	**Frame rate**	**Registration** **algorithm**
Voxelan (ERD)	Light section method using the line laser	1 camera unit	450 × 600 × 600 mm	1280 × 1024 px	2–15fps	Rigid
AlignRT (Vision RT)	Stereovision using a speckle pattern	1 to 3 cameras units (∼90°apart)	650 × 1000 × 350 mm	2048 × 2048 px	4–24 fps	Rigid
Catalyst (C‐RAD)	Structured light imaging	1 to 3 cameras units (∼120°apart)	1100 × 1100 × 2400 mm	640 × 480 px	8–24 fps	Deformable
IDENTIFY (Varian)	Stereovision using a speckle pattern	3 cameras units (∼90°apart)	500 × 500 × 400 mm	1280 × 1024 px	10 fps	Rigid

### 2.2 Position accuracy verification at Voxelan installation angle of 74 degrees

#### 2.2.1 Accuracy verification for translational movement

**FIGURE 3:**
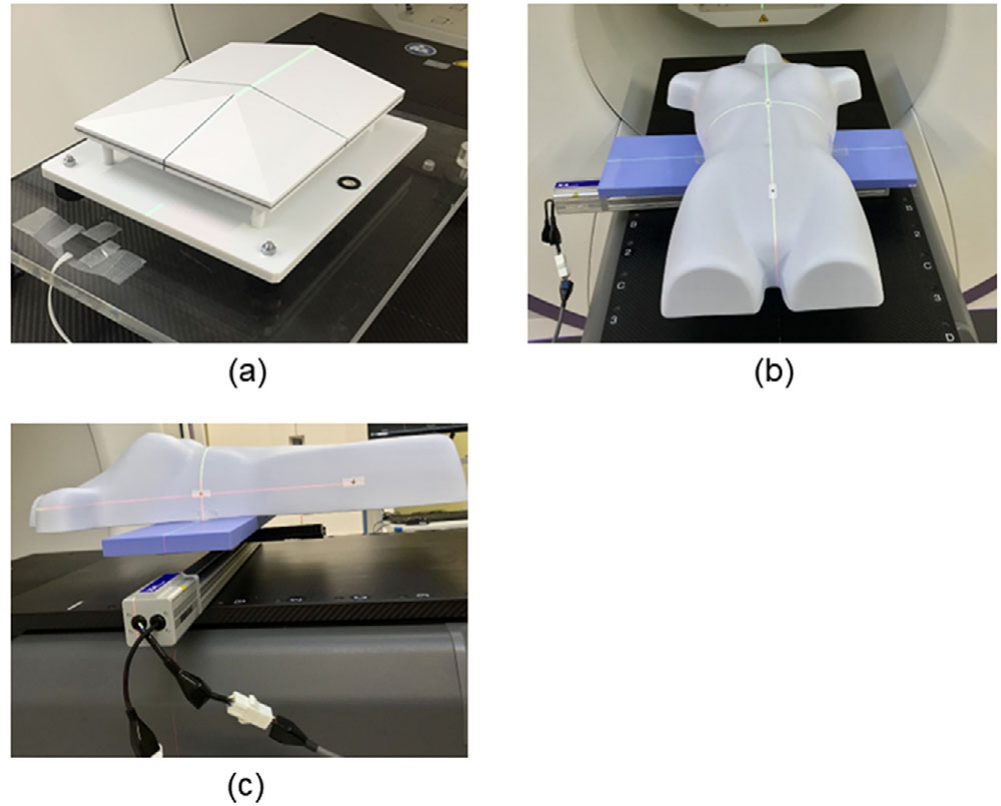
Devices for translational accuracy measurement: (a) Standard reference phantom, (b) torso phantom, and (c) actuator

The position accuracy of Voxelan at the installation angle of 74 degrees was verified using a standard reference phantom (ERD Co., Okayama, Japan) used in Voxelan's daily QA shown in Figure 3a and a torso phantom (Japan COPACK Co., Tokyo, Japan) used during Voxelan commissioning shown in Figure 3b. The Torso phantom is a plastic phantom that imitates a human torso created for clothing, and the position accuracy of the Voxelan was verified using the chest and pelvis. After setting the phantom so that the line of these phantoms and the virtual isocenter indicated laser pointer match, we obtained the surface reference. We then moved the phantom in the lateral (lat), longitudinal (lng), and vertical (vrt) directions individually in the range of ± 20 mm every 2 mm. The measurement time at each point was 10 s. We then measured the position with the Voxelan and the difference from the actual movement amount was recorded. A linear actuator XA‐42L‐400 (SUS Co., Ltd., Shizuoka, Japan) shown in Figure 3c was used to move the phantom, and the mean and standard deviation (SD) were calculated from the 10 scan measurements. The linear actuator has a resolution of 0.005 mm/pulse and a reproducibility of ± 0.02 mm.^9^ The Radixact's couch elevating function was used to move in the vrt direction. The resolution in the vrt direction of the Radixact's couch is 0.1 mm, and the reproducibility is ± 0.1 mm or less.

#### 2.2.2 Accuracy verification of rotational movement

**FIGURE 4:**
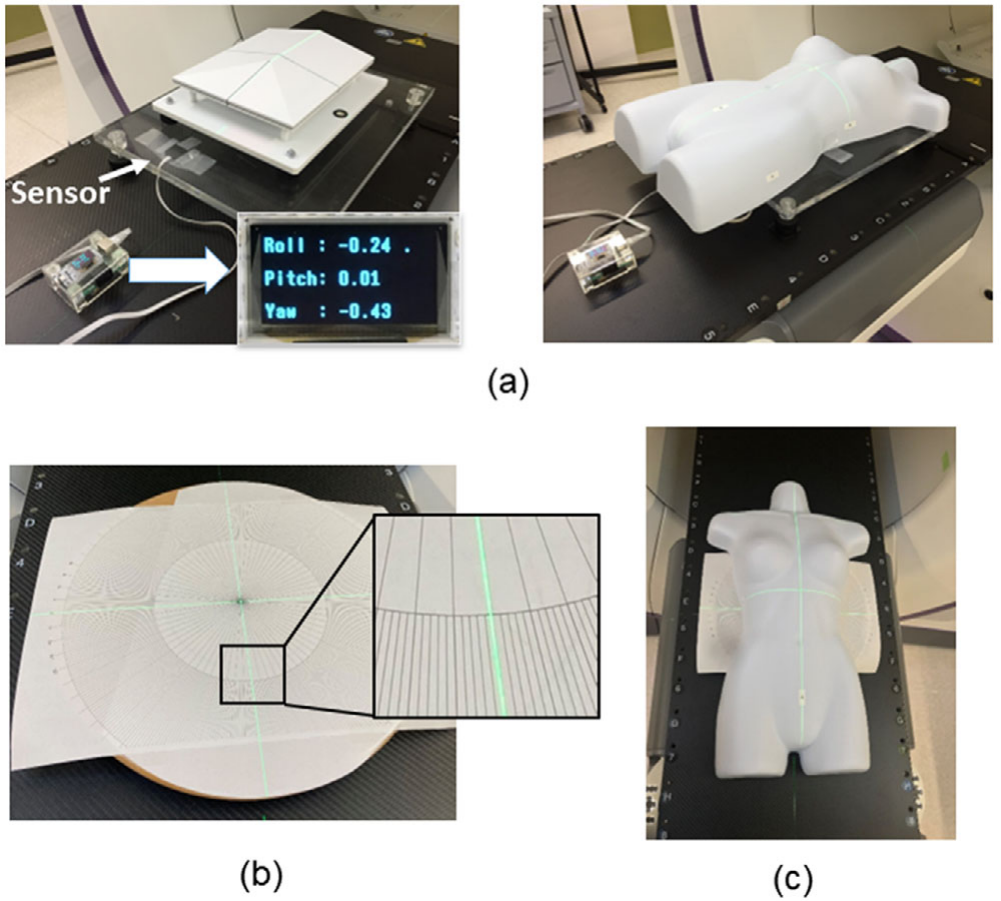
Devices for rotational accuracy measurement: (a) Measurement setup with Arduino‐based clinometer, (b) rotary table with hand‐made protractor, and (c) torso phantom mounted on rotary table

After installing the phantom in the same way as when verifying the accuracy of translational movement, and obtaining a surface reference, we verified the accuracy of the rotational movement of Voxelan at an installation angle of 74 degrees. An in‐house inclinometer using a 3‐axis acceleration/3‐axis gyro sensor module MPU‐6050 (InvenSenes Inc., CA, USA) and a HTIT‐WB32 microcomputer board (Heltec, Chengdu, China) was used to adjust the angles in the roll and pitch directions (Figure 4a). The rotation range in the roll and pitch directions ± 2 degrees, and the difference from the actual movement amount was recorded by measuring with Voxelan every 0.2 degrees. The resolution of the in‐house inclinometer is 0.01 degrees, and the reproducibility is ± 0.01 degrees or less.^10^ To adjust the angle in the yaw direction, a paper protractor was printed radially in 1 degrees increments and placed on a wooden rotary table (Figure 4b). Each phantom was placed on it to adjust the angle (Figure 4c). The resolution of the wooden turntable is 1 degree and the reproducibility is ± 1 degree or less. The rotation range in the yaw direction was ± 5 degrees, and the difference from the actual movement amount was recorded by measuring with Voxelan every 1 degree. The average value and SD were calculated from the 10 scan measurements at each measurement point. Measurements were made on the standard reference phantom and the torso phantom.

### 2.3 Inter‐fractional setup error for patients

**FIGURE 5:**
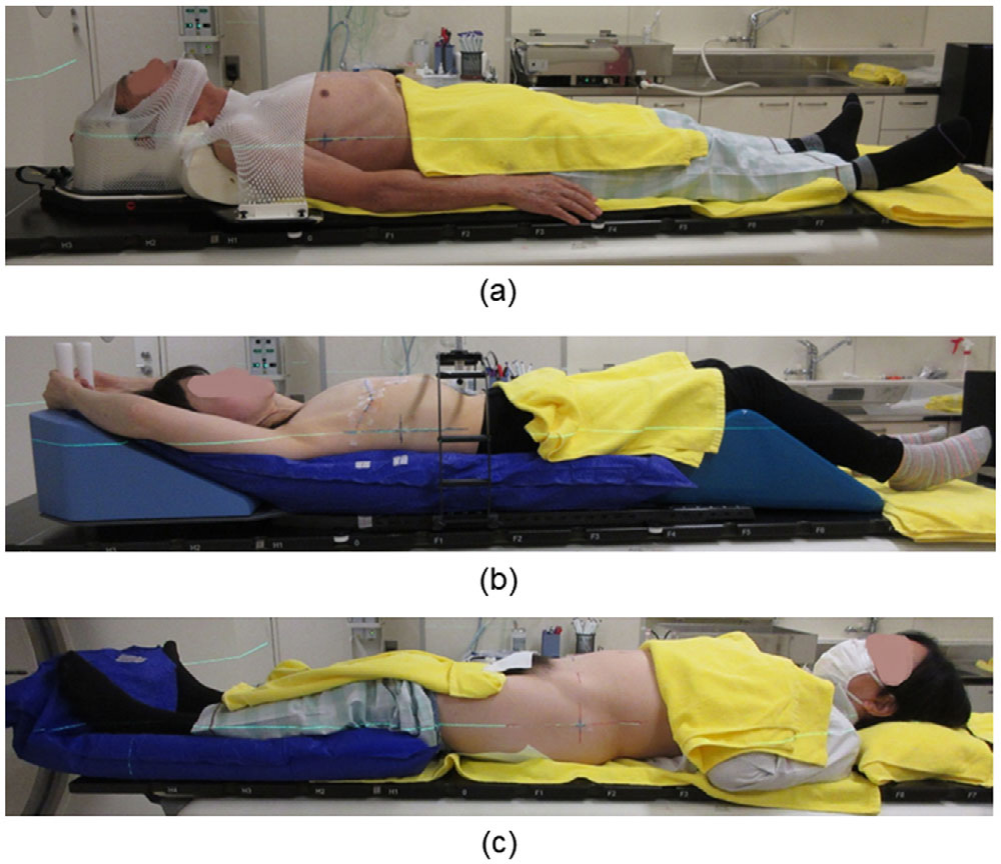
Patient positioning with immobilization device: (a) Head and neck, (b) chest, and (c) pelvis

We investigated the inter‐fractional setup error using Voxelan when radiotherapy was performed on head and neck, chest, and pelvis cases using different fixation methods for each site. Figure 5 shows the fixtures and postures for each treatment site. This study was approved (approval number: 2‐020054‐00) after submitting an ethics application to the in‐hospital ethics review committee in advance.

The fixture used for the head and neck cases was a Freedom LB‐SBRT (CDR Systems Corp, Alberta, Canada) as the base plate, and the pillow was a Silverman head support MTSILVER2B (Civco Medical Solutions, Iowa, USA) and INSTAFORM IFF03 (CDR Systems), the shell used was an open shell SL‐24‐S‐HYB‐O (CDR Systems) and a shell shoulder ST32‐01 (CDR Systems) with open eyes, nose, and lips (Figure 5a).

For chest patient cases, both hands were raised, and a Freedom LB‐SBRT, arm‐up module AU‐03 (CDR Systems), suction type patient fixation cushion Vac‐Q‐Fix RT‐4517‐70120F40 (Q‐Fix, USA), knee cushion ESF‐37 (Engineering System Co., Ltd. Nagano, Japan) were used for the fixture. In addition, a carbon fiber bridge SBRT‐B2 (CDR Systems) and a compression post (CDR Systems) were used to suppress respiratory movement (Figure 5b).

For the pelvic cases, a suction‐type patient fixation cushion Vac‐Q‐Fix RT‐4517‐70120F40 was used (Figure 5c).

The planning kVCT used Light Speed RT (GE Healthcare, Boston, USA). The imaging conditions were helical scan, 120 kVp, slice thickness 1.25 mm, helical pitch 1.5, matrix size 512 × 512, and FOV 500 mm. The images were taken under free breathing. After setting up at the virtual isocenter, the patient was moved to the isocenter on the Radixact couch and an MVCT was taken. Patient setup was performed so that the skin markers and the laser were matched on the odd‐numbered days of treatment (LS: laser setup), and the kVCT reference and the patient's position were matched using Voxelan on the even‐numbered days (SS: surface setup). The pelvic cases used the surface reference at the time of free exhalation obtained at the time of first acquisition. The reason for this is that the kVCT reference is affected by respiratory fluctuations in the umbilical region, which is a guideline for setup in the lng direction. Voxelan's ROI was set to include the feature points of the body surface such as shoulders and PTV so as not to include clothes. With reference to the image difference display between the body surface data and the reference data, the setup was performed so that the reference and actual lines match. Considering the patient's respiratory fluctuation and setup time, the position error measurement result indicated by Voxelan was set within ± 2 mm in each direction of lat, lng, and vrt. The LS was performed with Voxelan turned off, and the SS was performed so as to avoid setup bias due to the skin markers. Table 2 shows the acquisition conditions for MVCT. The imaging range was set to include the PTV, and the size of the bladder was also used only in the case of prostate, so the range was set to include the PTV and the bladder.

**TABLE 2:** Scan conditions for MVCT acquisition

**lng length of PTV**	**Algorithm**	**Pitch**	**Interval**
≦15 cm	IR general	normal	2 mm
>15 cm	IR general	coarse	3 mm

Inter‐fractional setup error analysis was performed offline using the review registration function of Precision Ver 2.0 (Accuray Inc., Sunnyvale, CA). Since the range cannot be specified in an automatic registration and the result changes depending on the conditions,^11^ the final position error calculation method was a manual registration of bone matching using six axes of lat, lng, vrt, pitch, roll, and yaw. Data was obtained from 19 patients with 10 to 12 fractions per patient for the head and neck case (LS : *n* = 241, SS : *n* = 226), 7 patients with 4 to 12 fractions per patient for the chest case (LS : *n* = 96, SS : *n* = 128), and nine patients with 5 to 20 fractions per patient for the pelvis case (LS : *n* = 241, SS : *n* = 189). The average value and SD of the 6‐axis inter‐fractional setup error of LS and SS were calculated and compared between LS and SS. Welch's *t*‐test was performed using IBM® SPSS® statistics Ver. 21 (IBM, New York, USA) for comparative analysis. In addition, the Vector offset (V_off_) of the inter‐fractional setup error was calculated using the following Equation (1), and the three‐dimensional position error was compared and examined.^12^

(1) $$V_{\mathrm{off}}=\;\sqrt{lat^{2}+lng^{2}+vrt^{2}}$$

Here, lat is defined as an inter‐fractional setup error in the lat direction, lng is defined as an inter‐fractional setup error in the lng direction, and vrt is defined as an inter‐fractional setup error in the vrt direction.

There are reports that set the margin when the minimum CTV dose is greater than 95% of the administered dose in 90% of the total sample,^13^, ^14^ as well as reports that it is appropriate to set the V_off_ shown when the cumulative probability is 90% of all samples as a margin.^12^ Therefore, we also used the value of the vector offset at cumulative probability of 90% as a comparative index in this study.

In addition, since registration in Radixct is performed on the entire body with kVCT and MVCT images, it has been reported that residual rotation errors sometimes cannot be corrected due to body changes in posture and anatomy.^7^, ^15^ In order to evaluate this residual rotation error, we analyzed the registration of head and neck cases (9 patients with hypopharyngeal cancer, LS: *n* = 76, SS: *n* = 73) by dividing them into cervical spine level (C1‐C3) and thoracic spine level (Th1‐Th3). Here, the rotation error of the inter‐fractional setup error registered at the cervical spine level is defined as Cervical, the rotation error of the inter‐fractional setup error registered at the thoracic spine level is defined as Thoracic, and the difference in Thoracic based on the Cervical is defined as the C‐T angle. Welch's *t*‐test and *F*‐test were performed using SPSS statistics Ver. 21 for comparative analysis of the C‐T angle in LS and SS.

### 2.4 Patient initial setup time and number of re‐setups

**FIGURE 6:**
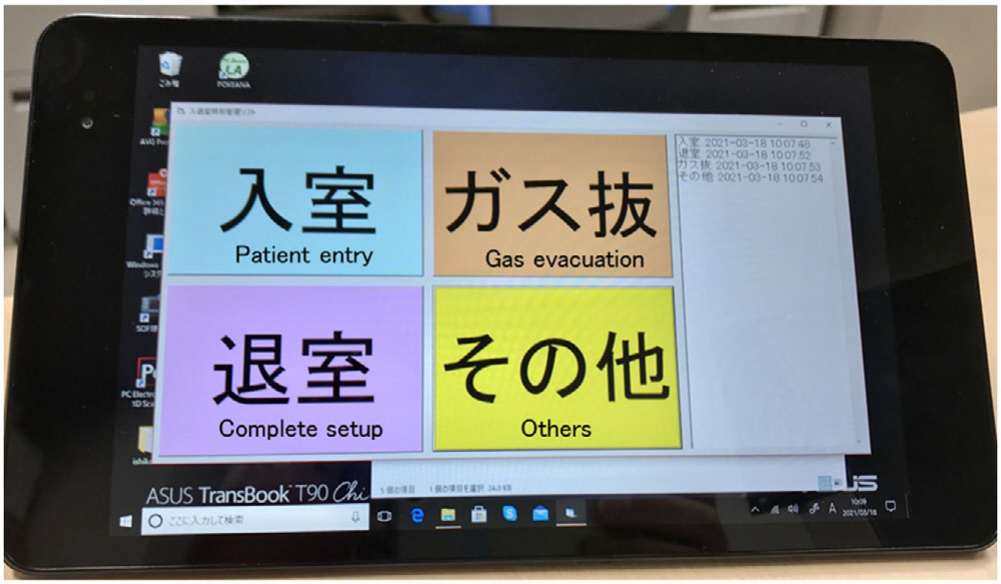
In‐house software for recording action events (patient entry, complete setup, gas evacuation, others). The events were automatically recorded by touching a button on the tablet device

In order to compare the time required for the initial setup between LS and SS, the patient's entry time and initial setup completion time were recorded. A tablet‐type Windows PC was installed at the entrance of the treatment room, and in‐house software was used to record the date and time of the event that was easily recorded by tapping the event item on the screen (Figure 6). The initial setup time was the time from when the patient entered the room until the radiologist completed the setup and returned to the operation room. The number of re‐setups was also recorded. For MVCT registration, re‐setup was performed when the anatomical landmarks and gross tumor volume in the clinical target volume deviated from the PTV, or when the risk organ deviated from the planning organ at risk.

## 3 RESULTS

### 3.1 Phantom position detection accuracy

**FIGURE 7:**
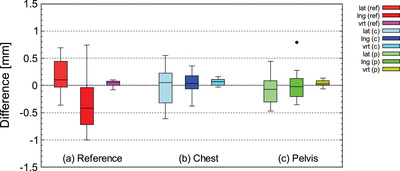
Differences of measured value by Voxelan from actual translational movement for the standard reference phantom (reference), chest of the torso phantom (chest), and pelvis of the torso phantom (pelvis)

**FIGURE 8:**
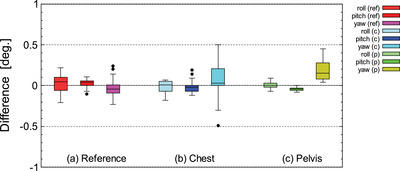
Differences of measured value by Voxelan from actual rotational movement for the standard reference phantom (reference), chest of the torso phantom (chest), and pelvis of the torso phantom (pelvis)

Figures 7 and 8 shows the difference between the actual amount of movement of the standard reference phantom and the torso phantom (chest, pelvis) and the measured values using Voxelan. The difference between the amount of movement and the measured value in the translational movement of all phantoms was 1 mm or less on average and the SD was 0.74 mm or less (Figures 7 and 8). In the rotational movement, the average value of the difference between the movement amount of all phantoms and the measured values was 0.50 degrees or less, and the SD was 0.27 degrees or less (Figures 7 and 8).

### 3.2 Inter‐fractional setup error for patients

**FIGURE 9:**
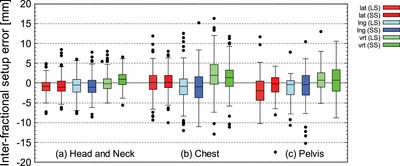
Inter‐fractional setup error in lat, lng, and vrt directions for head and neck case (a), chest (b), and pelvis (c)

**FIGURE 10:**
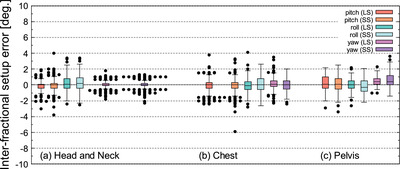
Inter‐fractional setup error in pitch, roll, and yaw directions for head and neck case (a), chest (b), and pelvis (c)

**FIGURE 11:**
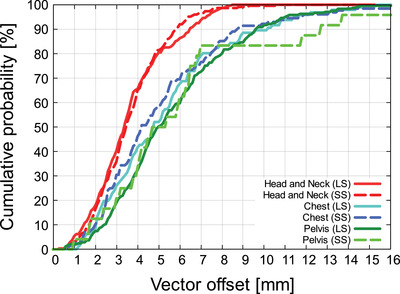
Cumulative probability of the vector offsets

Figures 9a and 10a shows the 6‐axis inter‐fractional setup error when LS and SS were performed for head and neck cases. Figure 11 shows the V_off_ for the cumulative probability distribution when LS and SS were performed for head and neck cases. The mean values of LS and SS of inter‐fractional setup errors based on MVCT did not show any significant difference in Welch's *t*‐test (*P* > 0.05) for all six axes (Figures 9, 10). In the V_off_ with a cumulative probability of 90%, the LS was 6.5 mm, while the SS was 5.8 mm (Figure 11).

**FIGURE 12:**
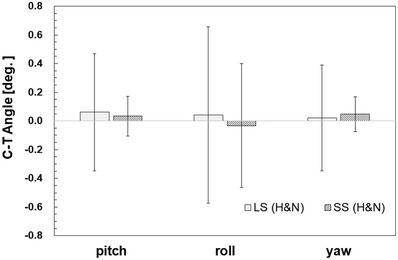
C‐T angles (pitch, roll, yaw) for head and neck cases (*n* = 9)

Figure 12 shows the results of the C‐T angle based on MVCT. The mean values of C‐T angle for LS and SS were not significantly different by Welch's *t*‐test (*P* > 0.05) in each rotation direction, but the SD was significantly smaller in SS than in LS in pitch and yaw (*F*‐test: *P* < 0.01).

Figures 9b and 10b shows the 6‐axis inter‐fractional setup error and the V_off_ for the cumulative probability distribution when LS and SS were performed for pelvis cases. Figure 11 shows the V_off_ for the cumulative probability distribution when LS and SS were performed for pelvis cases. The mean values of LS and SS of inter‐fractional setup errors based on MVCT did not show any significant difference in Welch's *t*‐test (*P* > 0.05) for all six axes (Figures 9 and 10). In the V_off_ with a cumulative probability of 90%, the LS was 9.6 mm, while the SS was 8.6 mm (Figure 11).

Figures 9c and 10c shows the 6‐axis inter‐fractional setup error probability distribution when LS and SS were performed for pelvis cases. Figures 11 shows the V_off_ for the cumulative probability distribution when LS and SS were performed for pelvis cases. The mean values of LS and SS of inter‐fractional setup errors based on MVCT did not show any significant difference in Welch's *t*‐test (*P* > 0.05) for all six axes (Figures 9 and 10). In the V_off_ with a cumulative probability of 90%, the LS was 9.6 mm, while the SS was 12.6 mm (Figure 11).

### 3.3 Patient initial setup time and number of re‐setups

Table 3 shows the time required for the initial setup and the number of re‐setups. In the cases of head and neck and chest, the average time was shorter for SS than for LS, and the SD also tended to decrease. In pelvic cases, the average time was shorter in LS, and the SD showed the same tendency. However, Welch's *t*‐test (*P* > 0.05) showed no significant difference between LS and SS at all sites.

**TABLE 3:** Comparison of initial setup time and number of re‐setups between laser setup (LS) and surface setup (SS)

**Initial setup time**				**Number of re‐setups**	
**Body part**	**LS**[sec]	**SS**[sec]	***P*‐value**	**LS**	**SS**
Head and neck	293 ± 212	282 ± 169	0.73	6	8
Chest	244 ± 168	221 ± 121	0.47	1	0
Pelvis	276 ± 106	294 ± 104	0.29	1	1

## 4 DISCUSSION

The accuracy of Voxelan at an installation angle of 74 degrees was confirmed with a standard reference phantom and torso phantom. As shown in Figures 7 and 8, the average value of the difference between the amount of movement and the measured value was 1 mm or less at all measurement points in the translation direction. The results met the criteria within 2 mm of the daily QA static localization test value published in the American Association of Physicists in Medicine Task Group report No. 147 (AAPM TG‐147).^16^ In addition, the SD of translational movement was 0.74 mm or less, which satisfied the reproducibility value of commissioning of AAPM TG‐147 of 1 mm.^16^ The results met the criteria within 1 mm of the static localization accuracy value published in the American Association of Physicists in Medicine Task Group report No. 302 (AAPM TG‐302).^17^ In the vrt direction of all phantoms in Figure 7, the difference between the actual movement amount and the measured value of the Voxelan was smaller than that in the lat and lng directions due to the different resolution in the vrt direction. As mentioned before, the lat and lng directions of Voxelan are 1.5 mm, while the resolution in the vrt direction is 0.02 mm, which is 64 times higher in resolution.^18^ The difference between the actual movement amount and the measured value of Voxelan in the vrt direction was smaller than that in the lat and lng directions, which was the same tendency even at the installation angle of 45 degrees.^18^

Next, the patient's inter‐fractional setup error was considered for each site. In the cases of the head and neck, there was no significant difference in the mean difference of the inter‐fractional setup error between SS and LS in all directions of the six axes (Figures 9 and 10). On the other hand, V_off_ showed the same tendency in SS and LS, but V_off_ with a cumulative probability of 90% was smaller in SS (Figure 11). Furthermore, since the SD of pitch and yaw was significantly smaller in SS at the C‐T angle, it was shown that SS with the Voxelan installation angle of 74 degrees had an initial setup accuracy equal to or higher than that of LS (Figure 12). There was no significant difference in the time required for setup between LS and SS.

In the chest cases, there was no significant difference in the mean difference in inter‐fractional setup error between LS and SS in all directions of the six axes (Figures 9 and 10). The V_off_ with a cumulative probability of 90% was smaller in SS (Figure 11). In previous reports,^19^ SGRT was considered to be effective in the chest region, and although there was no significant difference in this study, the same tendency was obtained. On the other hand, a report by Gopan et al. using Align RT (VisionRT Ltd., London, UK) stated that the reproducibility around the shoulder and clavicle was reduced.^20^ It has been reported that the SGRT system has imaging limitations depending on the position and number of cameras.^17^ However, in the case of our hospital, the installation position of Voxelan is at a position that looks down from the ceiling with an installation angle of 74 degrees rather than the usual installation angle of 45 degrees. As shown in Figures 7 and 8, Voxelan is sufficiently accurate since the positional error could be detected, and the inter‐fractional setup error of the head and neck case and the thoracic case due to elevation did not decrease (Figures 9 and 10). There was no significant difference in the time required for setup between LS and SS. Even in the chest cases, it was confirmed that the SS at the installation angle of 74 degrees had the same or higher initial setup accuracy as the LS.

In the pelvis cases, the inter‐fractional setup error in SS tended to be smaller in lng and vrt than in LS, but there was no significant difference in the average value of the inter‐fractional setup error with LS in all directions (Figures 9 and 10). The V_off_ with a cumulative probability of 90% was greater in SS than in LS (Figure 11). The reason why the V_off_ of SS was larger than that of LS may be that Voxelan has less body surface information when scanning the pelvis. In the case of LS, skin markers are applied to the front and sides of the patient (Figure 5c), so the setup position in the lng direction can be determined regardless of the shape of the patient's navel. On the other hand, in our hospital, the area around the pubis is covered with gauze in consideration of the patient's privacy when irradiating the pelvis. Therefore, in SS, the umbilical region is a characteristic point when setting up. The shape of the umbilical region may have increased the variation of SS in the lng direction compared to LS (Figure 9), which affected the cumulative probability distribution of the V_off_. In this study, the kVCT reference should have been set in the same way as the head and neck case and chest case, but since the umbilical region could not be clearly visualized as a reference, the surface reference was set. Therefore, it cannot be simply compared with the results obtained in the head and neck and chest cases. There was no significant difference in the time required for the initial setup between LS and SS. In the pelvic cases, it was confirmed that SS with an installation angle of 74 degrees had the same initial setup accuracy as LS, but it was necessary to note that the variation increased in the lng direction. The reason for the larger variation in the pelvic region is presumably because pelvic cases do not have a characteristic area to define the lng position, such as the jaw and nose in head and neck cases. The number of re‐setups in all cases tended to be the same for LS and SS.

In LS, it is recommended to draw a long skin markers in order to improve the initial setup accuracy in the rotation direction such as pitch.^21^ At our hospital, skin markers are drawn at least 5 cm for lateral and vertical axes of the patient and at least 15 cm for longitudinal axis (Figure 5). This study showed that in all cases, the initial setup accuracy was equal to or better than the difference between the mean values of LS with long skin markers and SS without skin markers. In addition, it was shown that SD may be smaller in SS than in LS at the C‐T angle of the head and neck case because SS can make changes in posture and anatomy smaller.

In this study, we verified the accuracy when the installation angle of Voxelan was 74 degrees, but if the installation conditions are different, the results of this study may not always be applicable. In addition, although long skin markers were drawn at this hospital, LS may be able to reduce the inter‐fractional setup error depending on how the skin markers are drawn. However, for Radixact, which cannot correct the couch for pitch or yaw, it is significant to find that eliminating the skin markers does not adversely affect the number of re‐setups and the inter‐fractional setup error. On the premise that MVCT is taken with the Radixact and IGRT is performed, initial setup without skin markers can be realized in all parts. As a result, the social and psychological burden on the patient and the burden on the staff to maintain the skin markers can be reduced.

## 5 CONCLUSIONS

Since the Radixact is set up with a virtual isocenter, it was necessary to direct the center of the field of view of the Voxelan to the virtual isocenter. Since Voxelan with an installation angle of 45 degrees interferes with the moving laser attached to the Radixact, we verified the accuracy of Voxelan when installed at an installation angle of 74 degrees. The accuracy of Voxelan at an installation angle of 74 degrees met the performance standards of an SGRT device as recommended by AAPM‐TG147. In the initial setup of the patient, the SS when Voxelan is set at an installation angle of 74 degrees is as accurate as or better than the LS, and at the C‐T angle, SS may be able to set up SD smaller than LS.

## AUTHOR CONTRIBUTIONS

Fumihiro Sasaki and Masayori Ishikawa analyzed all experimental data and wrote the main manuscript text. Yuusuke Yamashita did all experiments and summarized the data. All authors reviewed the manuscript.

## CONFLICT OF INTEREST

The authors declare no conflict of interest.
